# High-resolution detection of chromosomal rearrangements in leukemias through mate pair whole genome sequencing

**DOI:** 10.1371/journal.pone.0193928

**Published:** 2018-03-12

**Authors:** Anh Nhi Tran, Fulya Taylan, Vasilios Zachariadis, Ingegerd Ivanov Öfverholm, Anna Lindstrand, Francesco Vezzi, Britta Lötstedt, Magnus Nordenskjöld, Ann Nordgren, Daniel Nilsson, Gisela Barbany

**Affiliations:** 1 Department of Molecular Medicine and Surgery, Center for Molecular Medicine, Karolinska Institutet, Stockholm, Sweden; 2 Laboratory Division Karolinska University Hospital, Clinical Genetics, Stockholm, Sweden; 3 Science for Life Laboratory, Department of Biochemistry and Biophysics, Stockholm University, Stockholm, Sweden; German Cancer Research Center (DKFZ), GERMANY

## Abstract

The detection of recurrent somatic chromosomal rearrangements is standard of care for most leukemia types. Even though karyotype analysis—a low-resolution genome-wide chromosome analysis—is still the gold standard, it often needs to be complemented with other methods to increase resolution. To evaluate the feasibility and applicability of mate pair whole genome sequencing (MP-WGS) to detect structural chromosomal rearrangements in the diagnostic setting, we sequenced ten bone marrow samples from leukemia patients with recurrent rearrangements. Samples were selected based on cytogenetic and FISH results at leukemia diagnosis to include common rearrangements of prognostic relevance. Using MP-WGS and in-house bioinformatic analysis all sought rearrangements were successfully detected. In addition, unexpected complexity or additional, previously undetected rearrangements was unraveled in three samples. Finally, the MP-WGS analysis pinpointed the location of chromosome junctions at high resolution and we were able to identify the exact exons involved in the resulting fusion genes in all samples and the specific junction at the nucleotide level in half of the samples. The results show that our approach combines the screening character from karyotype analysis with the specificity and resolution of cytogenetic and molecular methods. As a result of the straightforward analysis and high-resolution detection of clinically relevant rearrangements, we conclude that MP-WGS is a feasible method for routine leukemia diagnostics of structural chromosomal rearrangements.

## Introduction

Many of the genetic markers that are relevant for classification of leukemia subtypes and disease prognosis, result from somatic structural rearrangements, such as translocations, inversions, or deletions. Karyotype analysis, regarded as the gold standard genome wide screening method to detect structural rearrangements, has a limited resolution to detect structural rearrangements and requires leukemic cells to divide in culture, which can occasionally fail [1]. Thus, in many instances, karyotype analysis needs to be complemented with other molecular cytogenetic techniques to reliably ascertain or exclude, the presence of specific rearrangements mandatory in treatment protocols [2]. Consequently, the routine diagnostic workflow in clinical laboratories requires parallel or sequential analysis with different methods to achieve an accurate and complete genetic classification of the leukemia.

Massively parallel sequencing (MPS) technologies are not only dramatically expanding our understanding of genetic aberrations underlying leukemias [3, 4] they also offer great potential to be implemented in the clinical diagnostic laboratories and may eventually replace currently used methods. Until now the implementation of MPS in diagnostic laboratories characterizing leukemia samples has largely focused on the detection of single nucleotide variations in panels of genes through targeted ultra-deep sequencing [5]. Although some approaches include particular structural rearrangements [6], the use of genome-wide assays to detect structural rearrangements has been limited to the research setting. The detection of structural aberrations, still relies on cytogenetics, fluorescence in situ hybridization (FISH) and molecular methods. Mate pair whole genome sequencing (MP-WGS) is a promising method to detect structural abnormalities and has so far been primarily used to resolve germ line rearrangements [7–10] with limited, yet successful application to leukemia [11]. Costs are rapidly dropping and at the same time the software to detect relevant genetic events is quickly improving. Both these facts combined with the possibility to comprehensive and high-resolution genetic information make MPS an attractive option for routine diagnostic laboratories. In the present report, we explore the feasibility of using low coverage MP-WGS in combination with a straightforward analysis approach to detect recurrent leukemia-specific rearrangements of known prognostic importance and to replace current diagnostic procedures.

## Material and methods

### Patient samples

Ten bone marrow samples taken at the time of leukemia diagnosis at the Karolinska University Hospital were selected to include genetic aberrations of prognostic relevance based on cytogenetic and FISH analysis. Samples from patients diagnosed with ALL (n = 5) included one t(1;19), two cases with *ETV6/RUNX1* fusion, one *KMT2A* (former *MLL*) and one t(9;22) rearrangement; The AML cases (n = 3) included one t(8;21), one inv(16) and one t(15;17) rearrangement. Two CML cases with the t(9;22) rearrangement were also included in the study (Table 1). The aberrations were present in ≥79% of the cells in the samples. The ethics review board at the Karolinska Institutet approved this study and informed verbal consent was obtained from the patients or their guardians.

**Table 1:** Validated MP-WGS findings.

Patient	Karyotype	FISH	Breakpoint A	Gene A	Breakpoint B	Gene B
**ALL1**	46,XX,der(19)t(1;19)(q23;p13)	*TCF3* 92%	chr1:164687337	*PBX1*:intron 2 (NM_002585)	chr19:1617928	*TCF3*:intron 16 (NM_003200)
**ALL2**	46,XX,?del(12)(p12)	*ETV6/RUNX1* 90%	chr12:12030146	*ETV6*:intron 5 (NM_001987)	chr12:43901893	*ADAMTS20*:intron 3 (NM_025003)
			chr12:12030152	*ETV6*:intron 5 (NM_001987)	chr21:36268099	*RUNX1*:intron 2 (NM_001754)
			chr12:43901900	*ADAMTS20*:intron 3 (NM_025003)	chr21:36268060	*RUNX1*:intron 2 (NM_001754)
**ALL3**	46,XY	*ETV6/RUNX1* 90%	chr5:15854389	*FBXL7*:intron 2 (NM_012304)	chr14:36210018	*RALGAPA1*:intron 11 (NM_014990)
			chr5:15854673	*FBXL7*:intron 2 (NM_012304)	chr7:54167959	TNRC18*:intron 7 (NM_001080495)
			chr7:47988425	*PKD1L1*:upstream (NM_138295)	chr12:12036803	*ETV6*:intron 5 (NM_001987)
			chr12:12036886	*ETV6*:intron 5 (NM_001987)	chr21:36401877	*RUNX1*:intron 2 (NM_001754)
			chr14:36209672	*RALGAPA1*:intron11 (NM_014990)	chr21:36403102	*RUNX1*:intron 2 (NM_001754)
**ALL4**	46,XY,t(9;22)(q34;q11)	*BCR/ABL1* 79%	chr9:133642586–133643724	*ABL1*:intron 1 (NM_007313)	chr22:23553422–23553571	*BCR*:intron 1 (NM_004327)
**ALL5**	46,XY	*KMT2A* 90%	chr10:21987471	*MLLT10*:intron 14 (NM_004641)	chr11:118066347	*AMICA1*:intron 8 (NM_153206)
			chr10:21987954	*MLLT10*:intron 14 (NM_004641)	chr11:118351075	*KMT2A*:intron 6 (NM_005933)
			chr11:118066347	*AMICA1*:intron 8 (NM_153206)	chr11:118351075	*KMT2A*:intron 6 (NM_005933)
**CML1**	45,X,-Y,t(9;22)(q34;q11)	*BCR/ABL1* 99%	chr9:133625252	*ABL1*:intron 1 (NM_007313)	chr22:23634126	*BCR*:intron 14 (NM_004327)
**CML2**	46,XY,t(9;22)(q34;q11)	*BCR/ABL1* 97%	chr9: 133659845–133660061	*ABL1*:intron 1 (NM_007313)	chr22:23632192–23632623	*BCR*:intron 13 (NM_004327)
**AML1**	46,XY,t(15;17)(q24;q21)	*PML/RARA* 95%	chr15:74316044	*PML*:intron 3 (NM_002675)	chr17:38489296	*RARA*:intron 2 (NM_000964)
**AML2**	46,XY,inv(16)(p13q22)	*CBFB/MYH11* 92%	chr16:15815171	*MYH11*:intron 32 (NM_002474)	chr16:67132568	*CBFB*:intron 5 (NM_001755)
**AML3**	46,XY,t(8;21)(q22;q22)	*RUNX1/RUNX1T1* 98%	chr8:93080937–93080955	*RUNX1T1*:intron 1 (NM_004349)	chr21:36230327–36230495	*RUNX1*:intron 6 (NM_001754)

Validated genetic findings with coordinates for the different breakpoints as determined by MP-WGS and/or Sanger sequencing

*TNRC: trinucleotide repeat containing 18

### Mate pair whole genome sequencing

Libraries were prepared from 1 microgram of high molecular weight genomic DNA using Illumina's Nextera Mate Pair Sample Preparation Kit, according to the manufacturer’s instructions for a gel-free preparation of 2 kb effective insert size library (size distribution mode 2 kb). The libraries were sequenced on an Illumina HiSeq 2500 sequencer, 2x100 bp to an average raw coverage depth of 5x. Raw sequence reads were base-called using CASAVA RTA 1.18.

### Data analysis

Following Illumina guidelines for mate-pair post processing, adapter sequences were removed using Trimmomatic v0.32. [12]. The remaining pairs were aligned to the hg19 human reference genome sequence using bwa 0.7.4-r385 [13] and resulted in a 3x mapped coverage. The mapped reads were processed using locally developed software TIDDIT (https://github.com/vezzi/TIDDIT) [14], publically available under General Public License version 3.0, implementing a sliding window analogue of a previously published procedure [15].

A list of detected rearrangements was generated by FindTranslocations with a sliding window size of 10000, a min insert size of 1000, max 100000, outtie orientation and a minimum supporting pairs cutoff at 8 and other parameters at default. Non-unique events were filtered out using a set of 35 germ-line genomes investigated by the same method [16]. None of these 35 genomes harbored rearrangements in regions recurrently involved in leukemia investigated in our sample cohort. Links supporting events connecting areas with repetitive sequences, which were annotated using repeat tracks from the UCSC genome browser, were considered misalignment artifacts and not further investigated. The remaining events are presented as a list of putative leukemia-specific events and graphically as circle plots [17].

**Fig 1:**
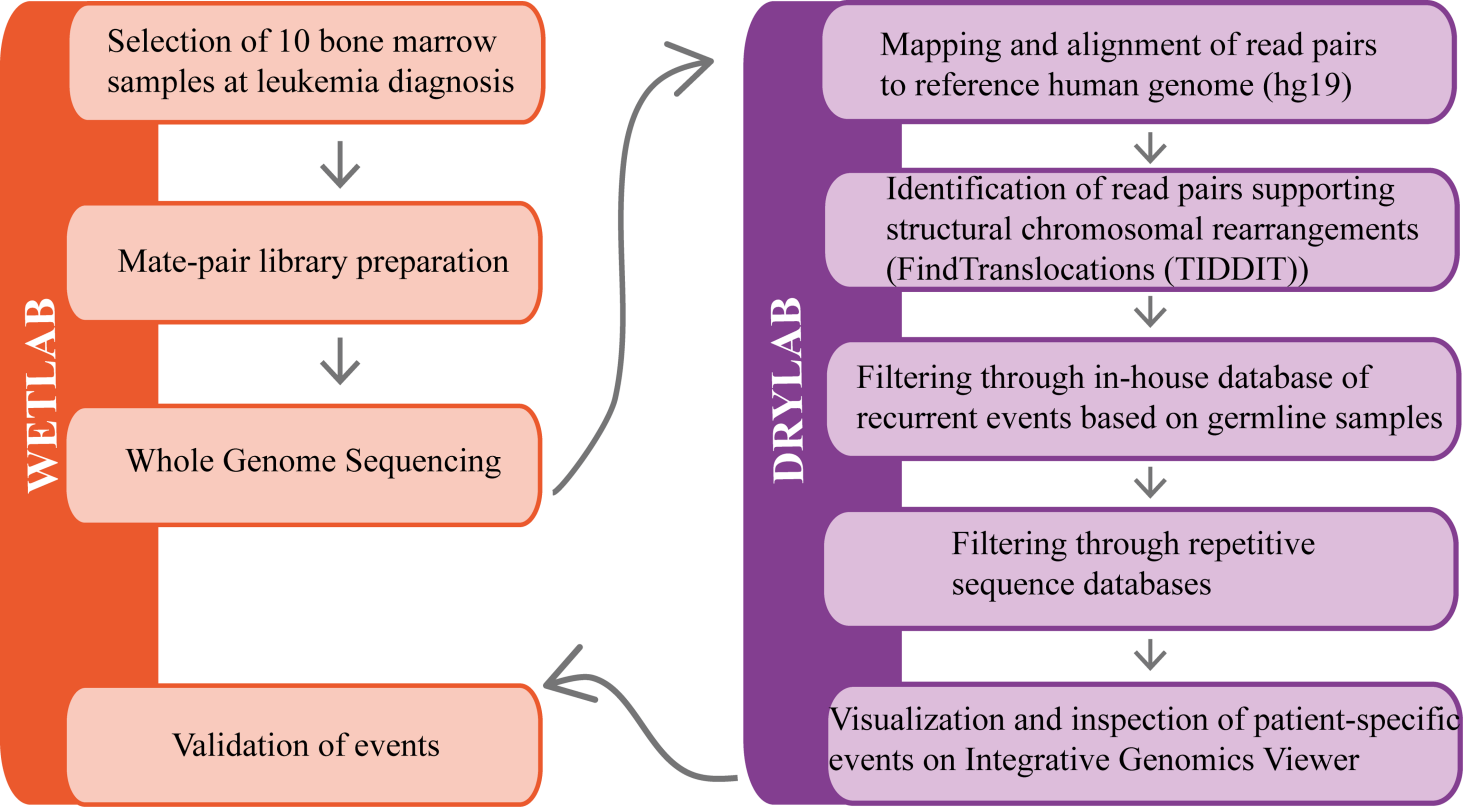
Schematic representation summarizing the workflow.

A schematic representation of the whole workflow is shown in Fig 1. All events were manually inspected at the nucleotide level using Integrative Genomics Viewer (IGV) [18]. Polymerase chain reaction and Sanger sequencing were used to validate unique events, with sufficient support from manual inspection that had escaped detection during routine analysis. Primer sequences and PCR conditions are described in the supplementary material, S2 Table and S1 Text.

## Results

**Fig 2:**
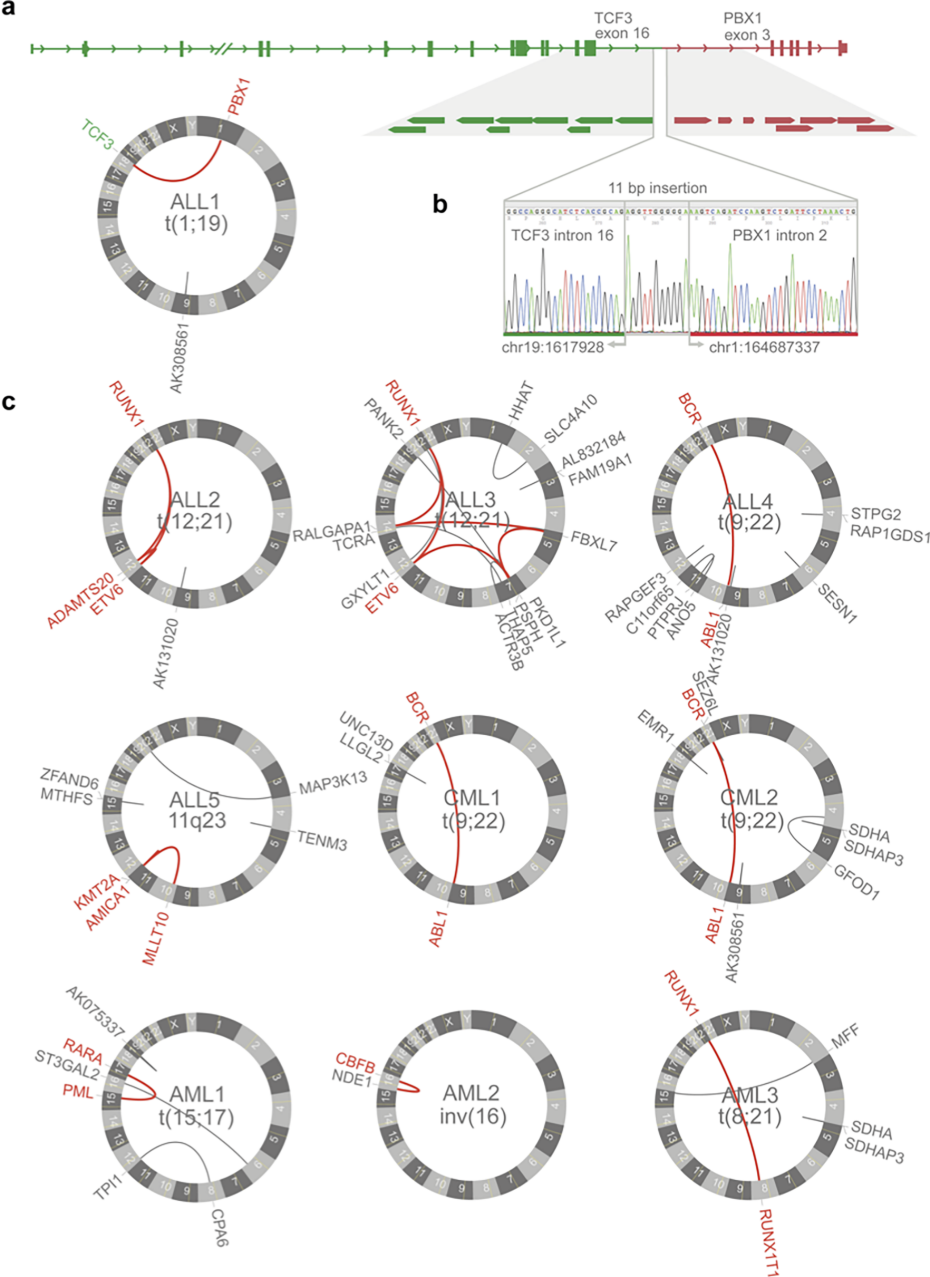
**(a) Graphic representation of MP-WGS events in ALL1.** Circos plot representing unique events detected by MP-WGS in ALL1 and graphic representation of *TCF3* (green) and *PBX1* (red) gene structure and the mate pair reads supporting the link between both genes. The red line in circos plot represents the event between *PBX1* (1q23) and *TCF3* (19p13.3). (**b)** Junction sequence of ALL1 Sanger sequence showing the 11 base-pair insertion at the junction between *TCF3* (green) and *PBX1* (red). **(c)** Circos plots of the remaining samples. Red lines: Unique events previously detected by FISH and/or karyotyping and novel unique events validated with Sanger; Gray lines: Unique events not further investigated.

All ten clinically relevant rearrangements, selected for this study, were successfully detected at high resolution through MP-WGS and our in-house analysis pipeline (Table 1). The ten recurrent fusion genes were unequivocally detected and we could identify the location of the breakpoints in all instances. Moreover, our analysis revealed additional aberrations (ALL3, ALL5) or complexity (ALL2) of the sought rearrangements that had escaped detection by previous cytogenetic analysis. Altogether, a total of 49 chromosomal events were detected by MP-WGS with four to five events on average per sample (range 1–14). The coordinates for all detected rearrangement breakpoints are given in Table 1 and in the S1 Table. All identified rearrangements are shown in Fig 2.

**Fig 3:**
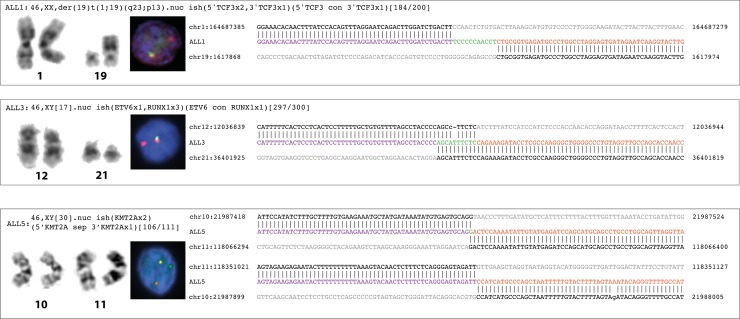
Karyotype, FISH and sequencing results. Karyotype and FISH results together with sequence composition at the junction region of the canonical fusion genes for samples ALL1, ALL3 and ALL5. Genome coordinates are based on hg19. Upper and lower sequences correspond to the reference sequences of the genes involved in the fusion. Patient sequences are shown in between in purple and orange. Insertions, deletions and microhomologies are shown in green.

The unbalanced t(1;19) in ALL1 was easily detected by MP-WGS that pinpointed the breakpoints to *PBX1* intron 2 and to *TCF3* intron 16, resulting in the expected *PBX1*/*TCF3* fusion gene (Figs 2A and 3, Table 1). Analysis of the junction sequence showed an 11 base-pair long non-templated insertion that was confirmed by Sanger sequencing (Fig 2B).

The cryptic t(12;21) rearrangement detected by MP-WGS in the t(12;21)-positive cases (ALL2, ALL3) but for both additional complexity was revealed. In addition to the expected *ETV6*/*RUNX1* fusion, which joined exon 5 from *ETV6* to exon 3 in *RUNX1*, the rearrangement in ALL2 also involved a third breakpoint located in chromosome region 12q12. The Zn-dependent protease gene *ADAMTS20* in this region (12q12) was involved in two novel fusion genes—*ADAMTS20*/*ETV6* and *RUNX1*/*ADAMTS20* (Fig 2C, Table 1). These chimeric genes are, however, not expected to result in fusion proteins since in both cases a stop codon is introduced downstream of the junction. ALL3 was found to harbor a very complex rearrangement involving several events on chromosome arms 5p, 7p, 12p, 14q and 21q (Figs 2C and 3, Table 1), all confirmed by Sanger sequencing. The pairs connecting these multiple breaks indicate that the events marked in red in Fig 2C must have occurred simultaneously. On derivative chromosome 21, exons 1 to 5 of *ETV6* were juxtaposed to exon 3 of *RUNX1*, creating the canonical fusion gene. We also found 10 base pairs of micro-homology in the junction between intron 5 in *ETV6* and intron 2 in *RUNX1* (Fig 3). Two of the remaining events connected genes on both paired chromosomes and resulted in two chimeric genes *RUNX1*/*RALGAPA1* on derivative chromosome 14 and *RALGAPA1*/*FBLX7* on derivative chromosome 5 (Fig 2C, Table 1). However, in both cases the partner genes are in opposite orientation and thus no functional chimeric protein can be generated.

Analysis of ALL5 showed that the *KMT2A* rearrangement consisted of an inverted insertion of a 0.29 megabase (Mb) fragment from 11q23.3 into the *MLLT10* locus at 10p12.31. As a consequence of the insertion, exons 1 to 5 from the *KMT2A* gene are juxtaposed to exons 15 through 24 of *MLLT10* in the same transcriptional orientation, thus creating the *KMT2A/MLLT10* fusion gene (Figs 2C and 3, Table 1). An interstitial deletion corresponding to 0.29Mb was found on 11q23.3 with no further fusion gene created that involved the remaining *KMT2A* exons.

The t(9;22) rearrangement in ALL4, CML1 and CML2 (Fig 2C) was found with MP-WGS. In ALL4, the *BCR* minor breakpoint juxtaposed exon 1 of *BCR* to exon 2 of *ABL1*, (Table 1) which resulted in the p190 *BCR-ABL1* fusion. On the other hand, the breakpoints in the CML cases were located as expected in the major breakpoint cluster region from *BCR* (intron 14 in CML1 and intron 13 in CML2) and in the first intron of *ABL1* (Table 1) and result in the p210 *BCR-ABL1* fusion characteristic of CML. In all three Philadelphia-positive cases the MP-WGS yielded a patient-specific junction sequence.

In AML1 case, the t(15;17)(q24;q11) reciprocal translocation used the bcr3 breakpoint of *PML* [19] and juxtaposed exon 3 of this gene to exon 3 of *RARA* creating the *PML/RARA* fusion gene (Fig 2C, Table 1). Similarly, in AML2 harboring the inv(16)(p13q22) the *CBFB/MYH11* chimeric gene was detected with a junction fusing exons 1 to 5 of *CBFB* to the exons downstream of intron 32 in *MYH11*, revealing an infrequent breakpoint on *MYH11* (Fig 2C, Table 1). The translocation t(8;21)(q22;q22) in AML3 produced the expected *RUNX1*/*RUNX1T1* chimeric gene (Fig 2C, Table 1), that joined exon 6 in *RUNX1* to exon 2 in *RUNX1T1*.

## Discussion

The major goal of this study was to evaluate the feasibility and suitability of MP-WGS in the diagnostic laboratory to detect clinically relevant, structural rearrangements in bone marrow samples from leukemia patients at diagnosis. For this purpose, we chose samples that were known to harbor known prognostic recurrent leukemia rearrangements in the majority of bone marrow cells.

In order to detect the leukemia–specific rearrangements, we limited our analysis to unique events in the sample cohort. We identified unique events by comparing each patient data against an in-house database of recurrent events. This in-house database consisted of events detected in germline samples and free from somatic events in the target regions investigated in the leukemia samples [16]. Events repeatedly detected in both cohorts were regarded as artifacts due to repetitive sequences, misalignment, polymorphic breakpoints or any other technical reason, and were filtered out. Discriminating between polymorphic breakpoints of germ-line origin and breakpoints of somatic origin is a potential problem that however is mitigated to an appreciable extent by the use of a germ line reference cohort. Alternatively, paired healthy tissue samples from the patients may serve, if a reference cohort is not available. However, obtaining a sample free from leukemic cells with good quality DNA is challenging at leukemia diagnosis. Also, the latter alternative significantly increases the costs.

We evaluated systematically each event based on the described criteria. Events that supported rearrangements between repetitive regions on both ends were disregarded whereas events where only one of the mates was in a repetitive region were retained. Though certain types of true structural rearrangements will certainly have breakpoints in repeat elements, the criteria allowed us to exclude putative false positives due to *in silico* alignment artifacts, while still retaining all the sought, clinically interesting events. Overall, the lists generated by this approach contained few sample-specific chromosomal events as seen in Fig 2C and in the S1 Table. Despite the filtering procedure the list contained a few recurrent events with similar breakpoints on one or even both sides. This was the case for chromosome 9 and linked primarily repetitive sequences to annotated genes of unknown function such as *AK308561* or *AK131020*. Also on chromosome 5 we found a recurrent link between *SDHA* and the pseudogene *SDHAP3*. Both types of events are likely artifacts due to misalignment not filtered out due to differences in the breakpoint coordinates.

Exact breakpoint recurrence cannot be used as a parameter to detect known rearrangements since most patients will have different coordinates for their leukemia-specific rearrangement and thus the rearrangement will likely present as unique when compared to previously analyzed cases. However, this issue can be easily circumvented in the diagnostic laboratory filtering the output against a list of candidate regions and genes in order to quickly confirm or exclude the sought clinically relevant rearrangements.

Though MP-WGS is primarily intended to detect and fine map specific rearrangements, it can also be used as a screening method in leukemia diagnostics since we have shown that it detects structural rearrangements present in a substantial proportion of cells. As suggested from this pilot study, MP-WGS is powerful to resolve complex rearrangements, such that both ALL2 and ALL3 cases showed additional breaks on other chromosome regions. This additional information however, does not affect the genetic classification of the leukemia. Moreover, MP-WGS made it possible to identify small insertions, deletions or microhomologies and the junction-specific sequence in some of the junctions. Microhomology and templated insertions have been observed during chromosome rearrangements mediated by replicative mechanisms [16]. In addition, these leukemia-specific sequences can be regarded as molecular markers to design patient-specific assays to monitor treatment response at the DNA level as shown by Meyer et al. [20].

MP-WGS, due to low coverage, is not suitable to detect single nucleotide variations (SNVs) and small indels as is the case for high coverage exome sequencing or paired-end whole genome sequencing. Also, detection of copy number alterations was beyond the scope of the present study for the same reason. While MP-WGS can be used as a high-resolution genome-wide screening method to identify structural alterations, gene panels with increased sequencing depth have been shown to be more suitable for the detection of somatic SNVs and indels with high sensitivity in myeloid malignancies. [5] [21].

In summary, we were able to clearly detect all clinically relevant somatic rearrangements in the patient samples. In addition, we could resolve and verify the unexpected complexity of particular rearrangements. Moreover, MP-WGS showed excellent resolution that allowed not only detection of the fusion genes but also the determination of the specific exons involved in the fusion proteins. An additional advantage of MP-WGS over conventional methods is that it also allows characterization of heterogeneous rearrangements as in the case for *KMT2A* gene fusions and directly provides information on partner genes in cases where several potential fusion partners have been described. Our results indicate that this method thus combines the genome-wide screening character of karyotyping together with the specificity and resolution offered by FISH and/or molecular methods. Together with a simple analysis approach implementing publicly available tools, MP-WGS is a feasible and attractive option to use in leukemia diagnostics.

## Supporting information

S1 Table Complete list of unique intra- and interchromosomal events. (DOCX)

S2 Table List of primers used in the validation of event breakpoints. (XLSX)

S1 Text Breakpoint validation procedure. (DOCX)
