# Supplementary material for: High-resolution detection of chromosomal rearrangements in leukemias through mate pair whole genome sequencing
Source: PLoS One. 2018 Mar 12;13(3):e0193928. doi: 10.1371/journal.pone.0193928 (PMC5846771; doi:10.1371/journal.pone.0193928)
Supplement: S1 Table — (DOCX) [file pone.0193928.s001.docx]

S1 Table: Complete list of unique intra- and interchromosomal events

| **Patient** | **Sought rearrangement**  **(% cells)** | **Breakpoint A** | **Gene/**  **Feature A** | **Breakpoint B** | **Gene/**  **Feature B** |
| --- | --- | --- | --- | --- | --- |
| **ALL1** | der(19)t(1;19)(q23;p13) | chr1:164687337 | *PBX1* | chr19:1617928 | *TCF3* |
|  | (92%) | chr9:66454988-66456578 | *AK308561* | chr9:68415086-68418863 | *rmsk_DNA* |
| **ALL2** | t(12;21)(p13;q22) | chr9:115245792-115248582 | *AK131020* | chr9:115480625-115482339 | *rmsk_SINE* |
|  | (90%) | chr12:12030146 | *ETV6* | chr12:43901893 | *ADAMTS20* |
|  |  | chr12:12030152 | *ETV6* | chr21:36268099 | *RUNX1* |
|  |  | chr12:43901900 | *ADAMTS20* | chr21:36268060 | *RUNX1* |
| **ALL3** | t(12;21)(p13;q22) | chr1:210813307-210822815 | *HHAT* | chr2:162499548-162501283 | *SLC4A10* |
|  | (90%) | chr3:67942661-67946769 | *AL832184* | chr3:68072911-68074851 | *FAM19A1* |
|  |  | chr5:4801781-4803213 | *rmsk_LINE* | chr7:56139899-56142348 | *PSPH/SUMF2* |
|  |  | chr5:15854389 | *FBXL7* | chr14:36210018 | *RALGAPA1* |
|  |  | chr5:15854673 | *FBXL7* | chr7:54167959 | *rmsk_LTR* |
|  |  | chr7:47988425 | *PKD1L1* | chr12:12036803 | *ETV6* |
|  |  | chr7:56107999-56108531 | *PSPH* | chr7:152540804-152543357 | *ACTR3B* |
|  |  | chr7:56168503-56171313 | *PSPH* | chr20:3880376-3882270 | *PANK2* |
|  |  | chr7:54169313-54169965 | *rmsk_DNA* | chr7:56174204-56178829 | *PSPH* |
|  |  | chr7:54170112-54177322 | *rmsk_SINE* | chr7:56173785-56177216 | *PSPH/CHCHD2* |
|  |  | chr7:108207296-108209809 | *THAP5* | chr14:22447800-22453404 | *TCRA* |
|  |  | chr12:12036886 | *ETV6* | chr21:36401877 | *RUNX1* |
|  |  | chr12:42477974-42479674 | *GXYLT1* | chr21:14968211-14970238 | *rmsk_SINE* |
|  |  | chr14:36209672 | *RALGAPA1* | chr21:36403102 | *RUNX1* |
| **ALL4** | t(9;22)(q34;q11) | chr4:99056404-99064292 | *STPG2* | chr4: 99299007-99309422 | *RAP1GDS1* |
|  | (79%) | chr6:109098690-109103919 | *rmsk_LINE* | chr6:109330042-109339000 | *SESN1* |
|  |  | chr9:115246643-115248800 | *AK131020* | chr9:115479978-115484779 | *rmsk_LTR* |
|  |  | **chr9:133642586-133643724** | ***ABL1*** | **chr22:23553422-23553571** | ***BCR*** |
|  |  | chr11:22262174-22268428 | *ANO5* | chr11:108313948-108317756 | *C11orf65* |
|  |  | chr11:47878756-47884444 | *rmsk_LTR* | chr11:48004893-48013624 | *PTPRJ* |
|  |  | chr12:48145854-48148858 | *RAPGEF3* | chr12:48314576-48318440 | *rmsk_LINE* |
| **ALL5** | 11q23 (KMT2A) | chr3:185173016-185173285 | *MAP3K13* | chr20:54856013-54862024 | *rmsk_LINE* |
|  | (90%) | chr4:183184552-183188383 | *TENM3* | chr4:183626145-183632533 | *TENM3* |
|  |  | chr10:21987954 | *MLLT10* | chr11:118351075 | *KMT2A* |
|  |  | chr10:21987471 | *MLLT10* | chr11:118066347 | *AMICA1* |
|  |  | chr11:118066347 | *AMICA1* | chr11:118351075 | *KMT2A* |
|  |  | chr15:80183435-80189107 | *MTHFS* | chr15:80422875-80437263 | *ZFAND6* |
| **CML1** | t(9;22)(q34;q11) | **chr9:133625252** | ***ABL1*** | **chr22:23634126** | ***BCR*** |
|  | (99%) | chr17:73570103-73572247 | *LLGL2* | chr17:73824254-73825521 | *UNC13D* |
| **CML2** | t(9;22)(q34;q11) | chr4:137213944-137214637 | *rmsk_SINE* | chr6:13486842-13489928 | *GFOD1* |
|  | 97% | chr5:244187-248242 | *SDHA* | chr5:1577188-1582121 | *SDHAP3* |
|  |  | chr9:66454940-66456419 | *AK308561* | chr9:68414779-68418695 | *rmsk_DNA* |
|  |  | **chr9:133659845-133660061** | ***ABL1*** | **chr22:23632192-23632623** | ***BCR*** |
|  |  | chr19:6889712-6893920 | *EMR1* | chr19:7104561-7107567 | *rmsk_SINE* |
|  |  | chr22:26427328-26429524 | *rmsk_LTR* | chr22:26592996-26599432 | *SEZ6L* |
| **AML1** | t(15;17)(q24;q21) | chr6:124077681 | *rmsk_LINE* | chr16:70451107 | *ST3GAL2* |
|  | (95%) | chr8:68374360-68376327 | *CPA6* | chr12:6978049-6979832 | *TPI1* |
|  |  | **chr15:74316044** | ***PML*** | **chr17:38489296** | ***RARA*** |
|  |  | chr19:22412653-22420665 | *rmsk_LTR* | chr19:28129889-28137332 | *AK075337* |
| **AML2** | inv(16)(p13q22) (92%) | **chr16:15815171** | ***MYH11*** | **chr16:67132568** | ***CBFB*** |
| **AML3** | t(8;21)(q22;q22) | chr2:228190071-228222401 | *MFF* | chr15:93835848-93842374 | *rmsk_LINE* |
|  | (98%) | **chr8:93080937-93080955** | ***RUNX1T1*** | **chr21:36230327-36230495** | ***RUNX1*** |
|  |  | chr5:233498-241941 | *SDHA* | chr5:1591770-1593317 | *SDHAP3* |

In red: breakpoints confirmed by Sanger sequencing or FISH only (bold characters); in black: PCR/Sanger validation failed; in grey: events not further investigated. Coordinates refer to the genome assembly hg19.
