# Supplementary material for: High-resolution detection of chromosomal rearrangements in leukemias through mate pair whole genome sequencing
Source: PLoS One. 2018 Mar 12;13(3):e0193928. doi: 10.1371/journal.pone.0193928 (PMC5846771; doi:10.1371/journal.pone.0193928)
Supplement: S1 Text — (DOCX) [file pone.0193928.s003.docx]

**Validation of Breakpoints**

**Whole Genome Amplification**

10 ng of genomic DNA and 10 microliters of 2x denaturation buffer incubated at 95°C for 3 minutes to denature template DNA. The reaction mix containing denatured template DNA was transferred into illustra^TM^ Ready-To-Go GenomiPhi V3 cake (GE Healthcare) and incubated for DNA amplification by Phi29 DNA polymerase at 30°C for 1.5 hours followed by 65°C for 10 minutes to stop the enzymatic reaction. Whole genome amplified DNA was diluted with TE for downstream PCR amplifications.

**Primer Design, Polymerase Chain Reaction and Sanger Sequencing**

The regions that were mapped by reads supporting the chromosomal events were used for primer design to validate the breakpoints. Primers with a size range of 20-25 bp were designed using default parameters of Primer3 software. Melting temperatures of the primers were ranging between 63-65°C and GC contents were in between 37.5% and 60%. Both forward and reverse primers had corresponding M13 sequences at their 5’ end. Primers sequences are listed in supplementary table S2.

100 ng of whole genome amplified DNA was mixed with 2.5 microliter of 10X PCR Buffer without MgCl_2_, 1.5 mM MgCl_2_, 0.2 mM dNTP, 0.2 micromolar forward and reverse primer and 2U of Platinum^TM^ *Taq* DNA Polymerase (Invitrogen). The reaction was incubated at 94°C for 2 minutes and followed by 35 cycles at 94°C for 30 seconds, 58-60°C (depending on the primer pair) for 30 seconds and 72°C for 30 seconds to 2 minutes (depending on the amplicon size). A final incubation of 2-5 minutes at 72°C was performed. PCR products were run on 2% agarose gel and then cleaned up for cyclesequencing using illustra ExoProStar 1-Step (GE Healthcare Life Sciences). Cyclesequencing reaction was performed using with BigDye Terminator v3.1 (Applied Biosystems) and M13 primers. After ethanol precipitation to clean-up unbound ddNTPs, fragments were sequenced in ABI 3730XL Genetic Analyzer (Applied Biosystems). Sequence fragments were then analysed on UCSC Genome Browser to determine coordinates at hg19 and sequence compositions at fusion sites.
